# Improved Activity against Acute Myeloid Leukemia with Chimeric Antigen Receptor (CAR)-NK-92 Cells Designed to Target CD123

**DOI:** 10.3390/v13071365

**Published:** 2021-07-14

**Authors:** Michael A. Morgan, Arnold Kloos, Daniela Lenz, Nadine Kattre, Juliette Nowak, Marco Bentele, Maximilian Keisker, Julia Dahlke, Katharina Zimmermann, Martin Sauer, Michael Heuser, Axel Schambach

**Affiliations:** 1 Institute of Experimental Hematology, Hannover Medical School, 30625 Hannover, Germany; lenz.daniela@gmx.de (D.L.); nowak.juliette@mh-hannover.de (J.N.); Marco.Bentele@stud.mh-hannover.de (M.B.); keisker.maximilian@mh-hannover.de (M.K.); dahlke.julia@mh-hannover.de (J.D.); zimmermann.katharina@mh-hannover.de (K.Z.); 2 REBIRTH Research Center for Translational Regenerative Medicine, Hannover Medical School, 30625 Hannover, Germany; 3 Department of Hematology, Hemostasis, Oncology and Stem Cell Transplantation, Hannover Medical School, 30625 Hannover, Germany; kloos.arnold@mh-hannover.de (A.K.); kattre.nadine@mh-hannover.de (N.K.); Heuser.Michael@mh-hannover.de (M.H.); 4 Department of Pediatric Hematology and Oncology, Hannover Medical School, 30625 Hannover, Germany; sauer.martin@mh-hannover.de; 5 Division of Hematology/Oncology, Boston Children’s Hospital, Harvard Medical School, Boston, MA 02115, USA

**Keywords:** alpharetroviral vector, acute myeloid leukemia, chimeric antigen receptor, CD123, interleukin 3 receptor subunit alpha, patient-derived xenograft, IL-15, NK-92

## Abstract

Anti-cancer activity can be improved by engineering immune cells to express chimeric antigen receptors (CARs) that recognize tumor-associated antigens. Retroviral vector gene transfer strategies allow stable and durable transgene expression. Here, we used alpharetroviral vectors to modify NK-92 cells, a natural killer cell line, with a third-generation CAR designed to target the IL-3 receptor subunit alpha (CD123), which is strongly expressed on the surface of acute myeloid leukemia (AML) cells. Alpharetroviral vectors also contained a transgene cassette to allow constitutive expression of human IL-15 for increased NK cell persistence in vivo. The anti-AML activity of CAR-NK-92 cells was tested via in vitro cytotoxicity assays with the CD123^+^ AML cell line KG-1a and in vivo in a patient-derived xenotransplantation CD123^+^ AML model. Unmodified NK-92 cells or NK-92 cells modified with a truncated version of the CAR that lacked the signaling domain served as controls. Alpharetroviral vector-modified NK-92 cells stably expressed the transgenes and secreted IL-15. Anti-CD123-CAR-NK-92 cells exhibited enhanced anti-AML activity in vitro and in vivo as compared to control NK-92 cells. Our data (1) shows the importance of IL-15 expression for in vivo persistence of NK-92 cells, (2) supports continued investigation of anti-CD123-CAR-NK cells to target AML, and (3) points towards potential strategies to further improve CAR-NK anti-AML activity.

## 1. Introduction

Retroviral vectors are commonly used to generate stably modified cells for preclinical and clinical applications. Gammaretroviral and lentiviral vectors are most often used, but experimental evidence also supporting the usefulness of the more recently engineered alpharetroviral vector systems has been steadily increasing, as a result of the close to neutral integration pattern [1,2]. Retroviral-mediated modification of immune cells, such as T and NK cells, with chimeric antigen receptors (CAR) has been used to improve treatment options for cancer patients, most prominently those with CD19^+^ lymphoid malignancies [3,4,5,6,7,8]. CARs are synthetic receptors that can be designed to target specific antigens (e.g., neoantigens or tumor-associated antigens). These artificial receptors typically consist of an antigen-binding domain, a hinge region, a transmembrane sequence, and an internal signaling domain that drives the cellular response upon recognition of the target antigen. The successful use of CAR-T and CAR-NK cells to treat CD19^+^ lymphoid cancers has served as the proof-of-concept to expand CAR therapies to treat other types of cancers. The majority of CAR-T-cell therapies is applied in autologous settings due to the risk of graft versus host disease (GVHD). As NK cells do not seem to cause GVHD even after mismatched allogeneic transplantation, CAR-NK cells may be more amenable to generation of off-the-shelf cell therapies [8].

Acute myeloid leukemia (AML) is the most common form of acute leukemia in adults and represents another disease setting in which CAR-T and CAR-NK cells may help improve patient outcomes [9]. AML is a biologically and molecularly heterogenous group of diseases and the long-term survival rate for most patients remains poor despite many efforts, including precision medicine approaches designed to target specific cellular pathways known to be dysregulated in leukemia cells [10,11]. The observation that, as compared to healthy hematopoietic cells, AML cells express higher levels of the surface antigen CD123 (the IL-3 receptor subunit alpha) makes this an interesting target for therapy [12,13]. For example, a humanized monoclonal antibody was developed to bind CD123 for induction of antibody-dependent T-cell-mediated cytotoxicity of AML cells and leukemia stem cells (LSC) [14]. CAR-T-cells designed to target CD123 showed anti-AML activity against leukemic blasts in vitro and in a xenogeneic mouse model that used the AML cell line KG-1a [15]. A multicenter phase I clinical trial (NCT04230265) to assess the universal CAR-T-cell platform (UniCAR) that was recently described to have efficient in vitro and in vivo activity against CD123^+^ AML is currently recruiting patients [16]. The UniCAR-T-cell system is controlled by interaction of an adaptor molecule (TM123), which binds CD123 and the extracellular CAR domain to activate UniCAR-T-cells upon recognition of CD123^+^ cells. Previously, we used in vitro cytotoxicity assays to show that anti-CD123-CAR-NK cells also had activity against KG-1a cells and primary human AML cells [17].

To test the anti-AML activity of anti-CD123-CAR-NK-92 cells, we generated self-inactivating (SIN) alpharetroviral vectors to constitutively express the anti-CD123-CAR, human IL-15, and enhanced green fluorescent protein (EGFP). As a control, a truncated version of the CAR that lacked the internal signaling domain was generated to evaluate the importance of CAR signal transduction after antigen-binding for anti-AML activity. Functionality of the vectors was shown as the modified cells stably expressed the anti-CD123-CAR and EGFP and secreted human IL-15. CAR-NK-92 cells effectively eliminated CD123+ AML cells in in vitro cytotoxicity assays and from the peripheral blood of a patient-derived xenotransplantation (PDX) model. The IL-15 expression cassette was crucial for in vivo persistence and anti-AML activity of NK cells.

## 2. Materials and Methods

### 2.1. Cell Lines

Human embryonic kidney 293T (#ACC 635; DSMZ, Braunschweig, Germany) and HT1080 fibroblasts (#ACC 315; DSMZ, Braunschweig, Germany) were cultured in Dulbecco’s modified Eagle’s medium containing stabilized glutamine (Biochrom, Berlin, Germany), 10% fetal bovine serum (FBS) (PAN Biotech, Aidenbach, Germany), 1% penicillin/streptomycin (P/S) (PAN Biotech, Aidenbach, Germany) and 1 mM sodium pyruvate (PAN Biotech, Aidenbach, Germany). The human natural killer cell line NK-92 (#ACC 488, DSMZ, Braunschweig, Germany) was cultured in Roswell Park Memorial Institute (RPMI)-1640 medium (PAN Biotech, Aidenbach, Germany) containing stabilized glutamine, 10% FBS, 1% P/S, 1 mM sodium pyruvate and 400 IU/mL human IL-2 (Proleukin S, Novartis Pharma GmbH, Nürnberg, Germany). The human acute myeloid leukemia (AML) cell line KG-1a (#ACC 421, DSMZ, Braunschweig, Germany) was cultured in Iscove’s modified Dulbecco’s medium containing stabilized glutamine (Biochrome, Berlin, Germany), 15% FBS, 1% P/S and 1 mM sodium pyruvate. Cell lines were routinely tested for mycoplasma infection.

### 2.2. Primary Human AML Cells and Xenotransplantation

Diagnostic white blood cells from peripheral blood of a 73-year-old male patient treated at Hannover Medical School were collected after obtaining informed consent according to the principles of the Declaration of Helsinki and the guidelines of the Hannover Medical School ethics committee (protocol #2613-2015). The patient was diagnosed with secondary AML, FAB subtype M5 and mutations in *ASXL1*, *FLT3*, *NF1*, *NPM1*, *RAD21*, *STAG1*, *TET2* and *TP53*. Briefly, mononuclear cells were isolated from peripheral blood by density centrifugation using Biocoll Separating Solution (Biochrom, Berlin, Germany) followed by depletion of CD3-positive cells using the CD3 MicroBeads kit (Miltenyi Biotec, Bergisch Gladbach, Germany). CD123 surface expression was determined by flow cytometry, and patient-derived xenotransplantation (PDX) models were generated by the successive passage of one million CD123-positive AML cells in six- to eight-week-old sublethally (2.5 Gy) irradiated female NOD.Cg-*Prkdc^scid^ Il2rg^tm1Wjl^*/SzJ mice transgenic for human interleukin-3, the granulocyte-macrophage-colony stimulating factor and stem cell factor (NSGS). Transplanted human AML cells and NK-92 cells were monitored by regular blood sampling and flow cytometry using a CytoFlex cytometer (Beckman Coulter, Krefeld, Germany). Isolated cells from bone marrow, spleen, and peripheral blood underwent erythrocyte lysis (BD Pharm Lyse, BD Biosciences, Heidelberg, Germany) and were stained to monitor engraftment and characterize immunophenotypes with anti-human antibodies CD45-Pacific blue (HI30, CAT 304021), CD33 PerCP-Cy5.5 (P67.6, CAT 366616) from BioLegend (Koblenz, Germany), CD123-APC (7G3, CAT 560087) and CD56-PE (B159, CAT 555516) from BD Biosciences (Heidelberg, Germany). Flow cytometry data were analyzed using FlowJo software (V10.0.7, TreeStar). NSGS mice were bred under pathogen-free conditions by the central animal laboratory of Hannover Medical School and animal experiments were approved by the Lower Saxony state office for consumer protection (Oldenburg, Germany) and were performed in accordance with the German animal protection law. Animal group size was calculated according to Charan and Kantharia [18].

### 2.3. Alpharetroviral Vector Constructs

**Figure 1:**
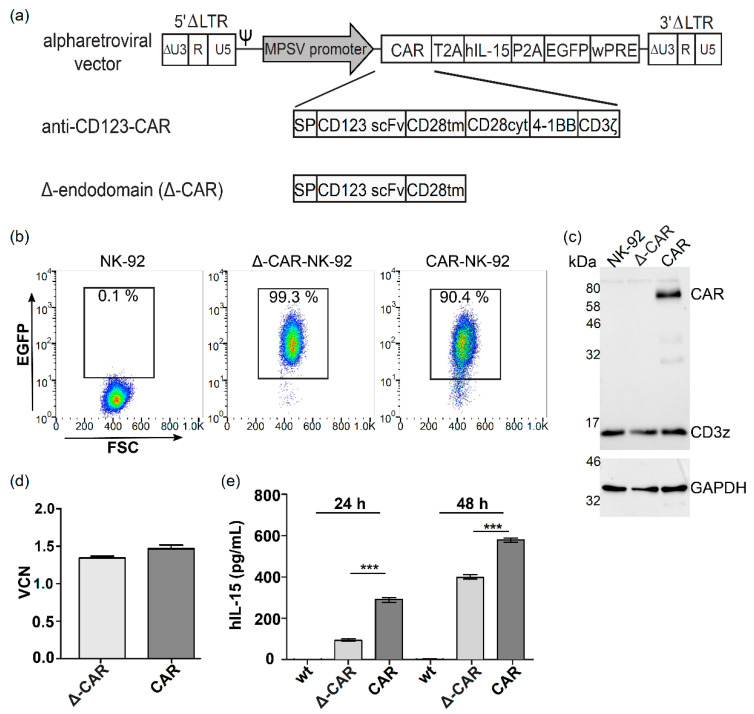
Alpharetroviral vectors were used to generate anti-CD123-CAR-NK-92 cells, which were also engineered to secrete hIL-15. (**a**) Schematic of alpharetroviral vectors. The myeloproliferative sarcoma virus (MPSV) promoter was used to express all constructs, including the full-length anti-CD123-CAR, the truncated CAR (Δ-CAR), human interleukin-15 (hIL-15), and enhanced green fluorescent protein (EGFP). SP, signal peptide derived from the GM-CSF receptor; CD28tm, the transmembrane domain of CD28; CD28cyt, the cytoplasmic domain of CD28; LTR, long-terminal repeat (ΔU3, R and U5); Ψ, packaging signal; wPRE, woodchuck hepatitis virus post-transcriptional regulatory element. (**b**) Flow cytometric analyses showing enrichment of anti-CD123-CAR-NK-92 cells and Δ-CAR-NK-92 cells using the EGFP marker transgene to detect modified cells. Representative results from more than four experiments are shown. (**c**) The intact anti-CD123-CAR was detected via immunoblot experiments with an antibody directed against CD3ζ, which also detects the endogenous CD3ζ visualized as the marked band just below 17 kDa. An antibody to detect GAPDH was used as an additional control. Representative immunoblots from two to three experiments are shown. (**d**) Quantitative PCR showed similar vector copy numbers in CAR-NK-92 and Δ-CAR-NK-92 cells. Shown are mean values with standard deviations from experiments accomplished as technical triplicates. (**e**) hIL-15 secretion from unmodified NK-92 cells (wt), Δ-CAR-NK-92 cells or full-length CAR-NK-92 was quantified by ELISA. Experiments were accomplished twice in triplicate. Mean values with ranges are shown. Statistically significant differences are indicated by *** (*p* ≤ 0.001).

A previously described CD123-specific third-generation CAR construct [19] was expressed from a self-inactivating (SIN) alpharetroviral vector containing a myeloproliferative sarcoma virus (MPSV) promoter and a woodchuck hepatitis virus posttranslational regulatory element (*wPRE*) (Figure 1a). The full-length CAR and truncated CAR (Δ-CAR), which lacks the internal signaling domain, both contained thosea asigna virus 2A (T2A) and porcine teschovirus-1 2A (P2A) sequences to allow concomitant expression of human IL-15 and EGFP, respectively. Cloning details are available upon request.

### 2.4. Alpharetroviral Vector Supernatant Production and Cell Modification

Calcium phosphate transfection of 293T-cells was used to generate alpharetroviral vector supernatants. For this, 5 µg of the alpharetroviral vector, 2.5 µg of the codon-optimized alpharetroviral gag/pol plasmid, and 2 µg of the RD114/TR envelope plasmid were applied per 10 cm cell culture dish (Sarstedt). Retroviral vector supernatants were collected at approximately 36 and 48 h after transfection, passed through Millex-GP 0.22 µm filters (Millipore, Schwalbach, Germany), concentrated by ultracentrifugation (13,238× *g*, ~16 h) and stored at −80 °C until use. To determine the titer of alpharetroviral vector productions, HT1080 cells were transduced with several different volumes of alpharetroviral vector supernatants by spinoculation (1 h, 400× *g*, ~35 °C) in the presence of protamine sulfate (4 µg/mL). NK-92 cells were transduced with known multiplicities of infection (MOI) using Retronectin (Takara Bio, Otsu, Japan), as described [19]. KG-1a cells were modified with the DsRed expressing vector, modified cells were enriched by sorting, and limiting dilution culture was performed to isolate single clones that stably express DsRed. Vector copy numbers (VCN) were determined using standard protocols. Briefly, genomic DNA was isolated from cells with the QIAamp DNA Blood Kit (Qiagen, Hilden, Germany). Vector copy number determination was performed by TaqMan quantitative PCR (ABI Taqman Fast Advanced Master Mix; Applied Biosystems, Life Technologies, Darmstadt, Germany) to detect the quantity of the woodchuck post-transcriptional regulatory element (*wPRE*) present in the integrated vector in relation to the amount of the host gene polypyrimidine tract-binding protein 2 (*PTBP2*), as previously described [20].

### 2.5. Immunoblot

Cell pellets were resuspended in lysis buffer (50 mM Tris-HCl, pH 7.5, containing 150 mM NaCl, 100 mM NaF, 1% Triton X-100, 1 mM Na_3_VO_4_, 1 mM dithiothreitol and protease inhibitors (cOmplete Mini, Roche Diagnostics, Mannheim, Germany), incubated 20 min on ice, centrifuged (15 min, 4 °C, 13,000× *g*), and supernatants were collected. The Coomassie dye-binding assay (Bio-Rad Laboratories, Hercules, CA, USA) was used to determine cellular protein concentrations. Equal total protein amounts were separated by SDS-PAGE and transferred to nitrocellulose membranes (GE Healthcare Life Science, Solingen, Germany). Membranes were blocked in Tris-buffered saline containing 5% (*w*/*v*) milk powder and incubated with antibodies (anti-CD3ζ-HRP (1:1000, Santa Cruz Biotechnology, CA, USA) and anti-GAPDH-HRP (1:10,000, GeneTex/BIOZOL, Eching, Germany). Membranes were washed, incubated with SuperSignal^®^ West Pico chemiluminescent substrate (ThermoScientific, Rockford, IL, USA), and images were captured with the Fusion imaging system (Molecular Devices, Biberach, Germany).

### 2.6. In Vitro Cytotoxicity Assay

The activity of anti-CD123-CAR-NK-92 cells was assessed in co-culture experiments with the CD123^+^ AML cell line KG-1a, which was modified with DsRed to simplify detection by flow cytometry. KG-1a-DsRed cells (0.05 × 10^6^) were seeded per well of a 24-well plate and cultured with unmodified NK-92 cells, truncated CAR-NK-92 cells, or intact CAR-NK-92 cells at effector-to-target (E:T) ratios of 1:1, 5:1, and 10:1 in a final volume of 1.1 mL RPMI culture medium containing 400 IU/mL hIL-2. Cells and supernatants were harvested directly after mixing (0 h time-point), and 24 and 48 h later. Effector and target cell ratios were analyzed by flow cytometry and the percentage of KG-1a-DsRed target cells at the 0 h time-point was set to 100% for comparison to the percentage of KG-1a-DsRed cells remaining after 24 and 48 h. Dead cells were excluded by DAPI (4′,6-diamidino-2-phenylindole) staining.

### 2.7. Cytokine Analyses

In vitro human IL-15 secretion by NK-92 cells was determined in supernatants harvested from cell cultures at 24 h and 48 h time-points. Cells (1 × 10^5^) were seeded in 700 µL of culture medium in duplicate wells of a 48-well plate, cells were pelleted by centrifugation (2.5× 10^3^× *g*, 5 min), and supernatants were harvested and stored at −20 °C until analyzed by enzyme-linked immunosorbent assay (ELISA) to quantify hIL-15 secreted by unmodified NK-92 cells, CAR-NK-92 cells, and Δ-CAR-NK-92 cells according to the manufacturer’s protocol (BioLegend^®^ Human IL-15 ELISA MAX™). To analyze a broader panel of cytokines secreted from CAR-NK-92 cells and Δ-CAR-NK-92 cells, the multi-analyte flow assay kit LEGENDplex™ (human CD8/NK panel)(BioLegend, CA, USA) was used as instructed to quantify secretion of IL-2, IL-4, IL-10, IL-6, IL-17A, TNF-α, soluble Fas (sFas), sFasL (ligand), IFN-γ, granzyme A, granzyme B, perforin and granulysin. Beads were acquired on a Beckman Coulter Cytoflex flow cytometer and analyzed with the LEGENDplex™ data analysis software. This method was used to assess in vitro cytokine levels in cell culture supernatants harvested from cytotoxicity assays at 24 h and 48 h time-points and in vivo cytokine production levels in peripheral blood acquired during routine determination of AML engraftment in mice prior to NK-92 cell application and at the time of sacrifice.

### 2.8. Statistical Analyses

GraphPad Prism (GraphPad Software, Inc, San Diego, CA, USA) was used to analyze and compare the activity of CAR-modified and control NK-92 cells. Two-way ANOVA with Bonferroni multiple comparisons was used to compare the cytotoxicity of CAR-NK-92 cells versus control NK-92 cells at different E:T ratios. Cytokine secretion levels were evaluated by an unpaired *t*-test with Welch’s correction for unequal variances. *p* values ≥ 0.05 were considered as statistically non-significant (n.s.), while *p* values ≤ 0.05 were considered significant and labelled by an asterisk (*). The Student’s unpaired *t* test was used to assess differences between groups for in vivo experiments.

## 3. Results

### 3.1. Modification of NK-92 Cells with Alpharetroviral CAR Vectors

Alpharetroviral constructs designed to express a third-generation anti-CD123-CAR via the MPSV promoter were modified to contain T2A and P2A sequences for constitutive concomitant expression of human IL-15 to improve NK cell persistence in vivo and EGFP to ease detection of modified cells (Figure 1a). Alpharetroviral particles were pseudotyped with the RD114/TR envelope glycoprotein and used to transduce NK-92 cells. Modified cells were enriched by flow-cytometric cell-sorting for EGFP expression, which resulted in over 99% purity of the truncated anti-CD123-CAR-NK-92 (Δ-CAR-NK-92) cells and over 90% purity of the full-length anti-CD123-CAR-NK-92 (CAR-NK-92) cells (Figure 1b). Expression of the full-length CAR was shown by immunoblots to detect CD3ζ that is expressed endogenously in NK-92 cells (band around 16 kDa, Figure 1c) as well as the CD3ζ that is fused with the CAR construct (band between 58 and 80 kDa, Figure 1c). In addition to the CAR construct, protein bands around 30 kDa and 35 kDa were also observed, which may indicate degradation products (Figure 1c). As expected, no signal was detected for the Δ-CAR construct as the CD3ζ sequence is part of the internal signaling domain that was deleted in this control construct. Analyses of vector copy numbers by quantitative PCR to detect the *wPRE* sequence of the alpharetroviral vector construct showed similar levels of alpharetroviral vector integrations in NK-92 cells transduced with Δ-CAR or the full-length CAR constructs (Figure 1d). Non-modified NK-92 cells were used as negative controls and showed no signal for *wPRE*, which indicated a vector copy number of zero, as expected (data not shown). To test for the ability of the modified NK-92 cells to produce and secrete human IL-15 (hIL-15), unmodified NK-92 cells, Δ-CAR-NK-92 cells, and CAR-NK-92 cells were cultured at similar densities, and supernatants were harvested and analyzed by ELISA, as described in the methods. These experiments showed that the hIL-15 expression cassettes functioned in the Δ-CAR-NK-92 cells and CAR-NK-92 cells, as supernatant hIL-15 levels rose from background levels in supernatants from unmodified NK-92 cells to average levels of 93 ± 3.5 pg/mL and 293 ± 8.6 pg/mL at 24 h for Δ-CAR-NK-92 cells and CAR-NK-92 cells, respectively (Figure 1e). Secretion levels of hIL-15 increased even further in the modified cells after 48 h in culture medium, with averages of 398 ± 7.4 pg/mL and 578 ± 9.8 pg/mL hIL-15 detected in supernatants from Δ-CAR-NK-92 cells and CAR-NK-92 cells, respectively, while hIL-15 levels in unmodified NK-92 cells remained below 2 pg/mL.

### 3.2. Improved Anti-AML Activity of CAR-NK-92 Cells In Vitro

**Figure 2:**
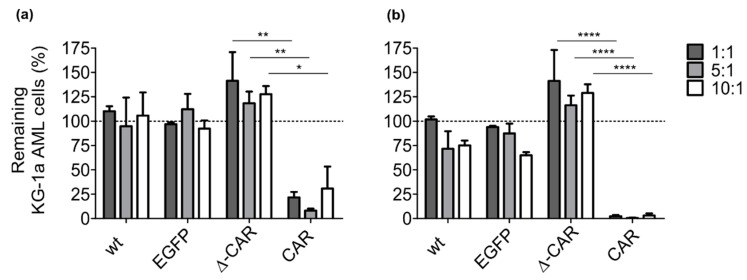
Co-culture experiments showed that CAR-NK-92 effector cells efficiently eliminated CD123^+^ KG-1a target cells. Different NK-92 cell groups (unmodified controls (wt), EGFP controls, Δ-CAR-, or CAR-modified) were tested for their activity against the AML cell line KG-1a. Co-cultures were accomplished at different effector:target (E:T) ratios (1:1, 5:1, 10:1) as indicated, and the loss of KG-1a cells was quantified at (**a**) 24 h and (**b**) 48 h after initiating co-culture experiments. KG-1a target cells were genetically modified to express the red flourescent protein DsRed to ease quantification by flow cytometry. Results from three experiments are shown. Mean values with standard deviations of KG-1a cells remaining after co-culture are given. Two-way ANOVA with Bonferroni multiple comparisons was used to compare cytotoxic activity. Statistically significant differences are indicated by * (*p* ≤ 0.05), ** (*p* ≤ 0.01), **** (*p* ≤ 0.0001). The dotted lines in the graphs serve as a reference for the percentage of KG-1a cells determined at initiation of the co-culture, which was set to 100%.

The anti-leukemia activity of unmodified NK-92 cells, NK-92 cells modified with an alpharetroviral vector to express only the EGFP marker as a control, Δ-CAR-NK-92 cells, and CAR-NK-92 cells against the CD123^+^ AML cell line KG-1a was compared in co-culture experiments. Three effector:target (E:T) ratios were tested (1:1, 5:1, 10:1) and loss of the KG-1a target AML cells was measured by flow cytometry at 24 h and 48 h (the gating strategy is shown in Figure S1). The CAR-NK-92 cells showed superior activity against the AML target cell line KG-1a as compared to unmodified NK-92 control cells, EGFP-NK-92 control cells, and Δ-CAR-NK-92 control cells (Figure 2). This was observed at all three different E:T ratios and at both time-points tested, with loss of more than 95% of AML cells after 48 h co-culture with CAR-NK-92 cells, while the target KG-1a cell population remained relatively constant or even expanded during co-culture with control NK-92 cells (Figure 2b).

### 3.3. CAR-NK-92 Cells Exhibit In Vivo Anti-AML Activity in an AML-PDX Model

**Figure 3:**
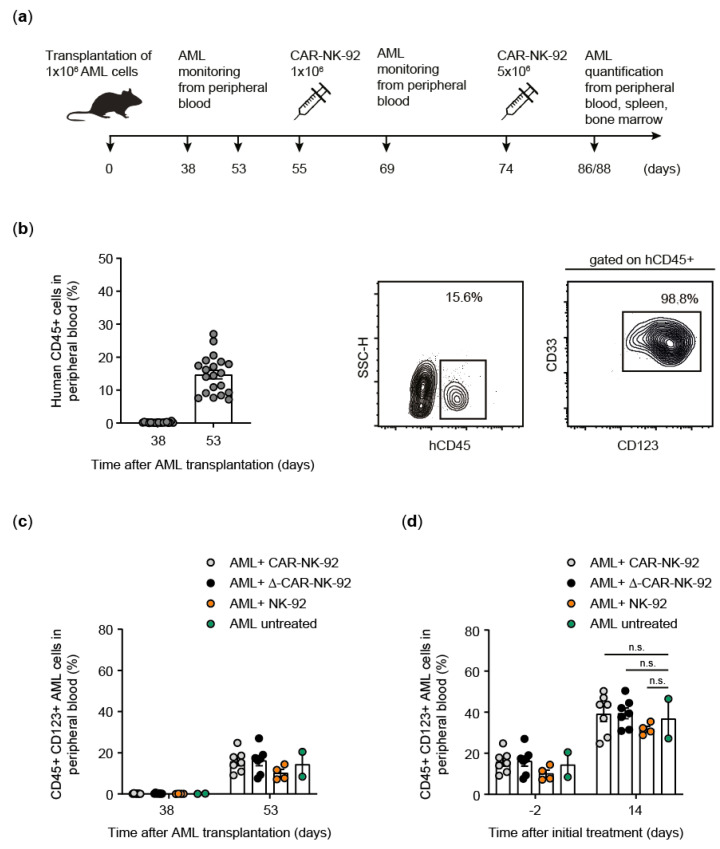
Establishment of an AML-PDX mouse model for anti-CD123-CAR-NK-92 treatment. (**a**) Experimental outline of in vivo AML-PDX engraftment and CAR-NK-92/effector cell treatment. (**b**) AML engraftment in peripheral blood at 38 and 53 days after transplantation. Representative flow cytometric analyses indicating primary human AML cells (hCD45^+^) and their stable phenotype (hCD45^+^CD33^+^CD123^+^) are shown. (**c**) Prior to treatment, AML-PDX mice were separated into four groups (untreated (*n* = 2), or treated with 1 × 10^6^ unmodified NK-92 cells (*n* = 4), treated with 1 × 10^6^ Δ-CAR-NK-92 cells (*n* = 7) or 1 × 10^6^ full-length CAR-NK-92 cells (*n* = 7)), each containing mice with similar AML levels as determined by flow cytometric analyses of peripheral blood (hCD45^+^CD123^+^). (**d**) After initial treatment, AML progression occurred in all groups as flow cytometric analyses showed an approximately two-fold increase in peripheral blood hCD45^+^CD123^+^ AML cells. Data are shown as mean ± standard error of mean (SEM). The unpaired *t*-test was used to assess differences between groups and *p* values ≥ 0.05 were considered statistically non-significant (n.s.).

**Figure 4:**
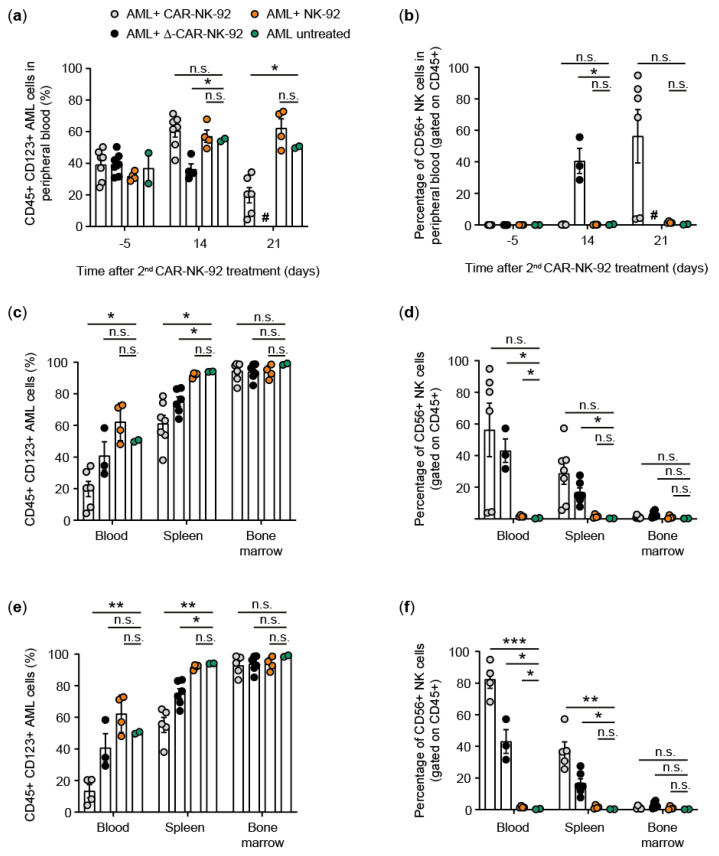
Anti-leukemic activity of CAR-NK-92 cells in vivo. (**a**) Reduction of AML blasts in peripheral blood following infusion of 5 × 10^6^ Δ-CAR-NK-92 or full-length CAR-NK-92 cells. # All Δ-CAR-NK-92 died or were sacrificed due to illness one week earlier. (**b**) Persistence of hIL-15-expressing Δ-CAR-NK-92 and CAR-NK-92 cells as measured by the hCD45^+^CD56^+^ population. (**c**) AML burden at time of sacrifice. AML blast percentages were determined in the peripheral blood, spleen, and bone marrow via flow cytometric analyses for hCD45^+^CD123^+^ cells. (**d**) NK cell persistence at time of sacrifice. NK-92 levels were determined in the peripheral blood, spleen and bone marrow via flow cytometric analyses for hCD45^+^CD56^+^ cells. (**e**) and (**f**) show the AML level and NK-92 cell persistence at the time of sacrifice as in panels **c** and **d**, respectively, but the two mice in the CAR-NK-92 group that showed no NK-92 persistence were excluded. Data are shown as mean ± standard error of mean (SEM) and the unpaired *t*-test was used to assess differences between groups. Statistically significant differences are indicated by * (*p* ≤ 0.05), ** (*p* ≤ 0.01), *** (*p* ≤ 0.001).

To further test the anti-AML activity of the IL-15-expressing CAR-NK-92 cells, a stable, transplantable AML-PDX model was generated by passaging and expanding CD123^+^ AML cells obtained from an AML patient during routine diagnostics at the time of diagnosis through NSGS mice. AML cells were then harvested from engrafted NSGS recipient mice, characterized by flow cytometry and cryopreserved until use. AML-PDX mice to test CAR-NK-92 activity were generated by intravenous infusion of 1 × 10^6^ AML cells per NSGS mouse. AML cells were allowed to engraft for several weeks and peripheral blood was periodically checked to determine the level of AML cells (human CD45^+^CD123^+^CD33^+^) as quantified by flow cytometry (Figure 3a). Seven weeks after infusion, mice displayed a mean AML burden of 15% (human CD45-positive cells co-expressing CD123 and CD33) in the peripheral blood (Figure 3b). The mice were divided into groups as shown in Figure 3c and treated with 1 × 10^6^ control NK-92 cells (unmodified or containing truncated CAR (Δ-CAR)) or with intact/full-length CAR-NK-92 cells via tail vein injection (Figure 3a). The distribution of AML blast levels among mice was similar among groups, with a slightly higher average disease burden in the Δ-CAR and CAR groups, as determined by flow cytometry of peripheral blood samples (Figure 3c). AML progression was observed in the two mice that were not treated with NK cells, but also in the other three experimental groups two weeks after the initial application of 1 × 10^6^ NK-92 cells, with the disease burden increasing from approximately 20% to around 40% AML blasts in peripheral blood, as quantified by flow cytometry (Figure 3d). Therefore, all mice in each of the three groups designated to receive NK-92 cells were administered an additional dose of 5 × 10^6^ NK-92 cells (unmodified, Δ-CAR or CAR, respectively) (Figure 3a, Figure 4).

After another two weeks, the amount of AML blasts in the peripheral blood increased to approximately 60% in the untreated control mice, as well as in the groups that were treated with unmodified NK-92 cells or the CAR-NK-92 cells (Figure 4a). At this time-point, several mice in the Δ-CAR-NK-92 cell control group died and the three remaining mice also had to be sacrificed due to poor health. In those animals for which the peripheral blood could be analyzed, the amount of AML blasts seemed to have remained stable at around 40% (Figure 4a). Analysis of NK cell persistence by measuring CD56 expression on hCD45^+^ cells also showed an average of about 40% NK cells in this peripheral blood population (Figure 4b). NK-92 cells were not detected in any of the other groups at this time-point. The remaining mice were sacrificed one week later. While AML blast levels were largely unchanged from the prior analysis one week earlier and remained at around 50–60% in the untreated control group and the group treated with unmodified NK-92 cells, the percentage of AML blasts in the group that received CAR-NK-92 cells was reduced from an average of around 60% to an average of about 20% (Figure 4a,c). Flow cytometric analysis to detect NK cells showed that approximately 60% (range: 3.7–96%) of hCD45^+^ cells in mice treated with CAR-NK-92 cells were CD56^+^, indicating that the anti-AML activity was associated with CAR-NK-92 cell persistence (Figure 4c,d). As evident from Figure 4b,d, CAR-NK-92 cells did not persist in two of the six mice that remained until this final time-point. Mean AML blast levels in the four mice with persistent CAR-NK-92 cells were 13% (range: 4–20%), as compared to 32% (range: 30–34%) in the other two mice in this group, which further supports the importance of NK cell persistence for anti-AML activity (Figure 4e,f). NK cells were not detected in the group that received unmodified NK-92 cells, indicating the necessity of the IL-15 expression cassette for in vivo NK cell persistence.

In addition to the peripheral blood, the activity against AML blasts in the spleen and bone marrow was also analyzed after sacrifice. In the untreated control animals and those treated with unmodified NK-92 cells, spleen infiltration by AML blasts was nearly complete with over 90% of CD45^+^CD123^+^ cells. The mice that were treated with Δ-CAR-NK-92 cells had about 20% lower AML cell infiltration than control groups, and those treated with CAR-NK-92 cells had the lowest splenic infiltration at an average of 60% (Figure 4c). This activity also correlated with the amount of Δ-CAR or CAR-NK-92 cells that were detected in the spleens via flow cytometry to quantify CD56^+^ cells in the hCD45^+^ population (Figure 4d,f). No reduction of AML blasts was observed in the bone marrow compartments of any of the groups, which was also reflected by a complete lack of NK cells in the bone marrow (Figure 4c–f).

### 3.4. Secretion of Degranulation Markers In Vivo

**Figure 5:**
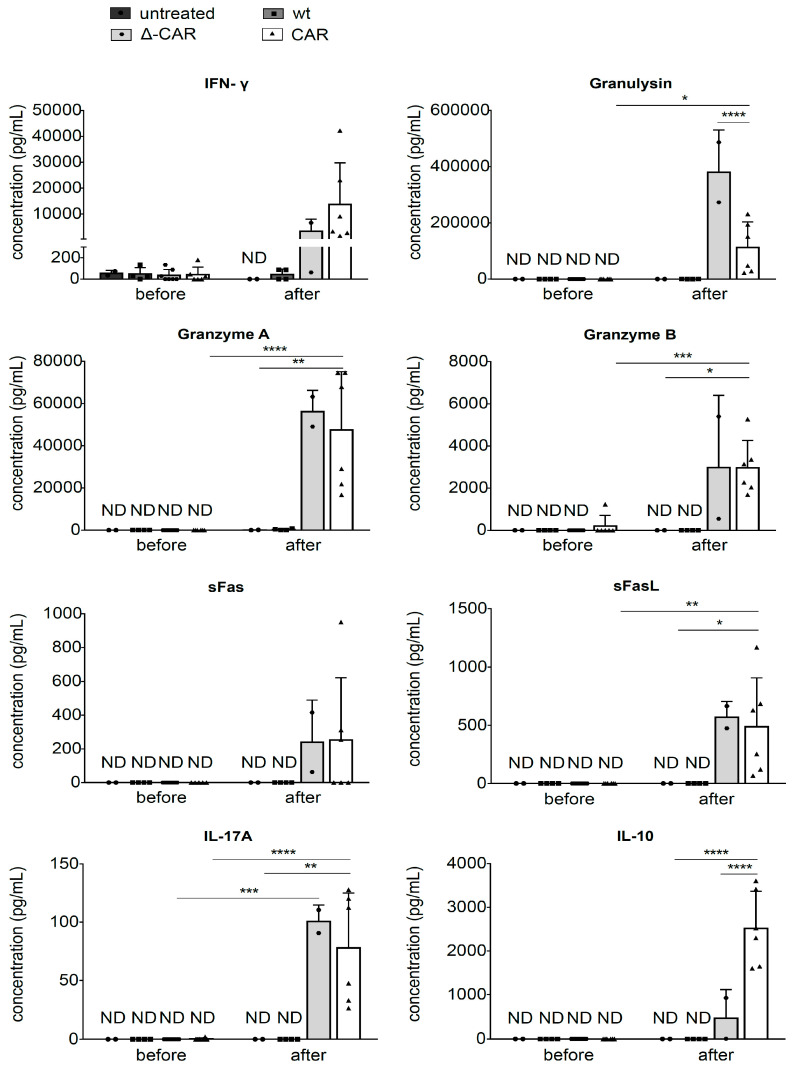
Quantification of secreted cytokines in the peripheral blood samples acquired from the mice analyzed in Figure 3 and Figure 4. Samples were processed according to the LEGENDplex™ human CD8/NK panel multi-analyte flow assay kit protocol and bead acquisition for cytokine quantification was accomplished with a Beckman Coulter Cytoflex flow cytometer. IFN-g, interferon-γ; sFas, soluble Fas; sFasL, soluble Fas ligand. Each data point represents the average cytokine amount from duplicate measurements of peripheral blood samples of an individual mouse. For measurements before NK cell application, 20 samples were evaluated: *n* = 2 mice for the untreated group, *n* = 4 mice for the unmodified (wt) NK-92 cell group, *n* = 7 for the Δ-CAR group and *n* = 7 for the CAR-NK-92 cell group. For measurements at time of sacrifice (after NK-92 treatment), *n* = 2 mice for the untreated group, *n* = 4 mice for the unmodified NK-92 cell group, *n* = 2 for the Δ-CAR group and *n* = 6 for the CAR-NK-92 cell group. Bars indicate mean ± SD of cytokine levels from all mice within a group. Two-way ANOVA with Bonferroni multiple comparisons was used to compare cytotoxic activity. Statistically significant differences are indicated by * (*p* ≤ 0.05), ** (*p* ≤ 0.01), *** (*p* ≤ 0.001), **** (*p* ≤ 0.0001). ND indicates below the level of detection.

To further evaluate the in vivo activity of CAR-NK-92 cells in comparison to that of unmodified NK-92 cells and Δ-CAR-NK-92 cells, cytokine secretion levels in peripheral blood samples acquired prior to administration of NK-92 cells and at the time of sacrifice were quantified using flow cytometry, as described in the methods section. Similar levels of IL-2, IL-4, IL-6, and TNF-α were observed in the four groups (untreated or treated with unmodified NK-92 cells, Δ-CAR-NK-92 cells, or CAR-NK-92 cells) both before and after infusion of NK-92 cells (data not shown). In contrast, several other cytokines known to serve as markers for NK cell activity and degranulation were strongly elevated in the peripheral blood samples collected at the time of sacrifice in mice treated with Δ-CAR-NK-92 cells or CAR-NK-92 cells (Figure 5). For example, IFN-γ levels were clearly increased in the groups of mice that received Δ-CAR-NK-92 cells or CAR-NK-92, however, this was not significantly higher than levels found in mice before NK-92 treatment. Granulysin expression was significantly higher in peripheral blood serum samples from mice that were treated with CAR-NK-92 cells as compared to before treatment, but was significantly lower than in mice that received Δ-CAR-NK-92 cells (Figure 5). Granzyme A, Granzyme B, sFasL, and IL-17A levels were significantly higher in the peripheral blood of mice treated with CAR-NK-92 cells versus those treated with unmodified (wt) NK-92 cells or from peripheral blood samples collected prior to CAR-NK-92 application (Figure 5). Although slightly elevated in mice that received Δ-CAR-NK-92 cells, IL-10 secretion was significantly higher in the peripheral blood of mice treated with CAR-NK-92 cells as compared to those treated with unmodified NK-92 cells or Δ-CAR-NK-92 cells (Figure 5). These results provide additional evidence that CAR- and Δ-CAR-NK-92 cells were functional in vivo and further confirm the importance of the hIL-15 expression cassette for in vivo persistence of the modified NK-92 cells.

## 4. Discussion

Adoptive immunotherapy approaches that incorporate CAR-NK cells have shown the potential to improve anti-cancer activity. In this study, we show that a self-inactivating (SIN) alpharetroviral vector system can be successfully used to deliver and stably express a CAR designed to recognize the AML target antigen CD123, hIL-15, and EGFP. NK-92 cells modified with the full-length CAR exhibited greater anti-AML activity in in vitro cytotoxicity assays and in an in vivo AML-PDX model as compared to unmodified or Δ-CAR-expressing NK-92 cells. Our data indicate that in vivo CAR-NK-92 cell persistence was necessary for the anti-AML activity and that hIL-15 expression was critical for in vivo CAR-NK cell persistence. These observations are consistent with earlier pre-clinical and clinical observations [8,21,22,23]. Infusion of IL-15 (2 µg/kg/d) into metastatic cancer patients led to greater than 350-fold expansion of CD56^bright^ NK cells that had enhanced tumor recognition, cytokine production, and cytotoxic activity [22]. Genetically engineering cord blood-derived anti-CD19-CAR-NK cells to co-express hIL-15 resulted in expansion and persistence of CAR-NK cells for at least 12 months, as shown by flow cytometric analyses of patient samples [8]. Additional evidence supporting the importance of IL-15 for success of adoptive immunotherapy was acquired in a clinical study that tested the efficacy of CAR-19 T-cells in CD19^+^ relapsed lymphoma patients. The authors found that high serum IL-15 levels were associated with higher numbers of CAR-19 T-cells in the peripheral blood, and that IL-15 and IL-10 peak serum levels were significantly higher in lymphoma patients who achieved complete or partial remission [6].

IL-10 levels were also significantly elevated in mice that received the CAR-NK-92 cells in comparison to those that received Δ-CAR-NK-92 cells or unmodified NK-92 cells. In addition to promoting NK cell in vivo persistence, IL-15 was also shown to induce IL-10 expression, further supporting the fact that our alpharetroviral gene transfer strategy was functional. Increased IL-10 in the serum of animals treated with CAR-NK-92 cells may have contributed to the better anti-AML activity in the peripheral blood as compared to mice that received the Δ-CAR-NK-92 cells. This is supported by earlier work that showed that IL-15-induced IL-10 led to increased cytotoxic activity of NK cells [24]. IL-10 was also shown to promote CD8^+^ T-cell anti-tumor activity and expansion of intratumoral CD8^+^ T-cells [25,26]. In contrast, other studies have shown that IL-10 secretion by regulatory T-cells can suppress murine NK cell cytotoxicity [27]. Additionally, impaired function of NK cells in AML patients at diagnosis was predictive for failure to achieve remission after chemotherapy, and was described to be caused by IL-10, as IL-10 levels in supernatants were directly correlated to the amount of NK cell dysfunction in NK-AML cell co-culture experiments [28]. An earlier study showed that CD137-CD137L (4-1BB-4-1BBL) interaction impairs NK cell anti-tumor activity in humans via induction of IL-10 and TNF release from AML cells, which allowed AML cells to evade being eliminated by NK cells [29]. Therefore, the cell source of IL-10, as well as additional intra- and intercellular signals may influence whether it activates or inactivates NK cell immune responses. Although the third-generation CAR used in our study had a 4-1BB costimulatory domain, it does not contain the extracellular ligand binding domain and, thus, is not expected to induce AML cells to secrete IL-10 or TNF.

We also detected elevated peripheral blood levels of other cytokines known to be involved in NK cell-mediated cytotoxicity. For example, IL-17A, sFas, sFasL, IFN-γ, granzyme A/B and granulysin levels were increased in the peripheral blood of mice treated with the CAR-NK-92 and Δ-CAR-NK-92 cells as compared to levels detected in mice prior to NK-92 administration or after infusion with unmodified NK-92 cells. The observation that in vivo levels of secreted degranulation markers was similar between the CAR-NK-92 and Δ-CAR-NK-92 groups may indicate the natural capacity of NK cells to become activated in the presence of cancer cells, in this case AML cells, regardless of whether the NK cells are modified with a fully functional CAR. These results may also reflect the cytotoxic nature of NK-92 cells and might be explained by the in vivo expansion of these cells due to the IL-15 expression cassette. In general, these observations indicated the in vivo persistence and activation of IL-15 expressing CAR-NK-92 and Δ-CAR-NK-92 cells, which is consistent with previous work that showed similar effects of IL-15, as discussed above. Comparison of peripheral blood hIL-15 levels among the different treatment groups would be another interesting method to assess in vivo persistence of the administered NK-92 cells. As one of the functions of NK cells is to detect and eliminate cancer cells, it was not unexpected that the Δ-CAR-NK-92 cells also showed some in vivo activity. However, the reduction of AML burden measured as the amount of peripheral blood AML cells was clearly greater in mice treated with CAR-NK-92 cells, at least after the second infusion of NK-92 cells.

The inability of the CAR-NK-92 cells to completely clear AML in the PDX model is at least partially due to the biodistribution of the CAR-NK-92 cells. CAR-NK-92 cells were readily detected in the peripheral blood (68–94% of hCD45^+^ cells) of four of the mice in this group, and the amount of AML cells decreased from about 60% to around 10% hCD45^+^CD123^+^ cells. It remains unclear why the CAR-NK-92 cells did not persist in the other two mice in this treatment group, but the peripheral blood AML cells were clearly higher in these two mice as compared to the four mice in which the CAR-NK-92 cells persisted (30–34% vs. 4–20%, respectively). Δ-CAR-NK-92 cells also persisted in the peripheral blood as 40% of hCD45^+^ cells were CD56^+^ in this group. However, in contrast to the persistent CAR-NK-92 cells, Δ-CAR-NK-92 cells only resulted in stabilization of AML levels (around 40% before and after application of the second Δ-CAR-NK-92 dose). Similarly, CAR-NK-92 cells also persisted in the spleens of treated mice, as 5–57% (average 28%) of hCD45^+^ cells were CD56^+^. Consistent with this persistence, there was an overall lower percentage of hCD45^+^CD123^+^ AML cells in the spleens of mice treated with CAR-NK-92 cells as compared to mice treated with unmodified NK-92 cells or Δ-CAR-NK-92 cells (61% vs. 91% or 75%, respectively).

In contrast to the peripheral blood and splenic compartments, our study showed a distinct lack of CAR-NK cell anti-AML activity in the bone marrow, which is likely due to the lack of NK cell migration into the bone marrow compartment as we did not detect the infused NK cells in the bone marrow. It is possible that the cells did transit to the bone marrow, but did not persist long enough to be detected at the time of sacrifice or to eliminate AML cells from the bone marrow. Results from others indicate that homing of expanded NK cells into the bone marrow may be inhibited due to increased C-C chemokine receptor type 5 (CCR5) expression and decreased C-X-C chemokine receptor type 4 (CXCR4) expression [30]. The same group showed improved bone marrow homing of NK cells in NSG mice via transfection with CXCR4^R334X^, a gain-of-function variant [31]. Incorporation of such strategies into the CAR-NK cells is expected to help improve elimination of AML cells, including leukemic stem cells, from the bone marrow. This may be an important concept in AML therapy as leukemia cells are known to occupy and remodel the bone marrow niche to favor leukemic cell survival over healthy hematopoiesis [10,32,33]. Strategies that contribute to eradication of medullary and extramedullary AML cells are expected to help improve patient outcome as disease relapse due to the inability of current treatment protocols to completely eliminate all AML cells remains one of the main reasons for the poor outcome of most AML patients [34].

Additional AML targets that have been investigated for CAR therapies include CD33 [35], CLEC12A [36], the high-affinity folate receptor beta [37], and B7-H3 [38]. Beyond adoptive cell therapeutic strategies, bi- and tri-specific killer engager (BiKE and TriKE, respectively) approaches appear promising as AML treatment [39]. For example, these were developed to promote formation of immunologic synapses between target cells that express CD33, which is expressed in myeloid cells, including AML, and CD16 on NK cells. The TriKE constructs were further designed to contain a human IL-15 crosslinker that prolonged in vivo persistence of adoptively transferred human NK cells and improved anti-cancer activity in a xenogeneic AML mouse model based on the AML cell line HL-60 [39].

An earlier AML-PDX study showed that a combination of NK-92 cells modified to express CD16 (CD16^+^NK-92) and application of an anti-CD123 monoclonal antibody (7G3) had anti-AML activity [40]. The authors applied irradiated CD16^+^NK-92 cells and the 7G3 antibody on days 3, 5, 7, 10, and 12 after infusion of the primary human AML cells, and this treatment resulted in a 7–10 day longer median survival as compared to control-treated mice [40]. Irradiated NK-92 cells were also used to treat cancer patients [35,41,42]. In our model, we monitored AML cell engraftment over several weeks to more closely recapitulate the clinical setting at diagnosis prior to application of NK-92, Δ-CAR-NK-92 or CAR-NK-92 cells. In earlier experiments, we also tried multiple doses of non-irradiated CAR-NK-92 cells (not carrying the hIL-15 expression cassette) to treat the engrafted AML in our PDX model, but did not observe any persistence of NK-92 cells or anti-AML activity (data not shown). Based on these results, we chose not to irradiate the NK-92/CAR-NK-92 cells in the current model. The earlier death of the Δ-CAR-NK-92 treated mice may have resulted from a combination of less efficient elimination of AML cells and expansion of Δ-CAR-NK-92 cells. This is supported by the higher percentage of AML cells and similar NK-92 cell persistence/expansion detected in the mice treated with Δ-CAR-NK-92 as compared to those treated with CAR-NK-92 cells. Thus, our observations indicate that future treatment strategies should explore irradiated CAR-NK-92 cells or translation of this approach to primary human NK cells. Optionally, a genetic safety switch for controlled removal of CAR-NK cells could be included for use in the case of adverse events. However, CAR-NK-92 cells engineered to target CD123 (IL3RA) may pose a risk of off-tumor toxicity as up to 9% of healthy hematopoietic progenitor cells also express CD123 [43].

Additional potential risks of gene-modified NK cells that constitutively secrete hIL-15 include systemic toxicity due to high hIL-15 serum levels and uncontrolled proliferation. However, continuous IL-15 secretion was not reported to be problematic in lymphoma patients treated with anti-CD19 CAR-NK cells that were engineered to constitutively express hIL-15 [8]. In contrast, uncontrolled proliferation of modified NK-92 cells may have been a contributing factor that led to the earlier death of the Δ-CAR-NK-92 group, which had to be sacrificed one week earlier than the CAR-NK-92 group (Figure 4). In summary, this work illustrates the potential of CAR-NK-92 cells redirected to target CD123^+^ AML cells in vitro and in vivo. Anti-AML in vivo activity required NK cell persistence, which was achieved using alpharetroviral vectors to engineer NK cells to constitutively co-express hIL-15 in addition to the anti-CD123-CAR. Additional modifications of CAR-NK cells to increase bioavailability in specific target organs or niches are expected to further increase the anti-AML activity of this targeted immunotherapeutic approach. Until these limitations can be properly addressed, including demonstration of anti-AML activity in the tumor microenvironment, this approach may be constrained to use as a strategy to bridge the time until transplantation in AML patients.

## Data Availability

Data is contained within the article and supplementary material.

## Supplementary Materials

The following are available online at https://www.mdpi.com/article/10.3390/v13071365/s1, Figure S1: Representative dot plots showing a typical co-culture experiment.
