# Supplementary material for: Improved Activity against Acute Myeloid Leukemia with Chimeric Antigen Receptor (CAR)-NK-92 Cells Designed to Target CD123
Source: Viruses. 2021 Jul 14;13(7):1365. doi: 10.3390/v13071365 (PMC8310147; doi:10.3390/v13071365)
Supplement: Supplementary file 1 [file viruses-13-01365-s001.zip › viruses-1269001-supplementary.pdf]

Supplementary Materials

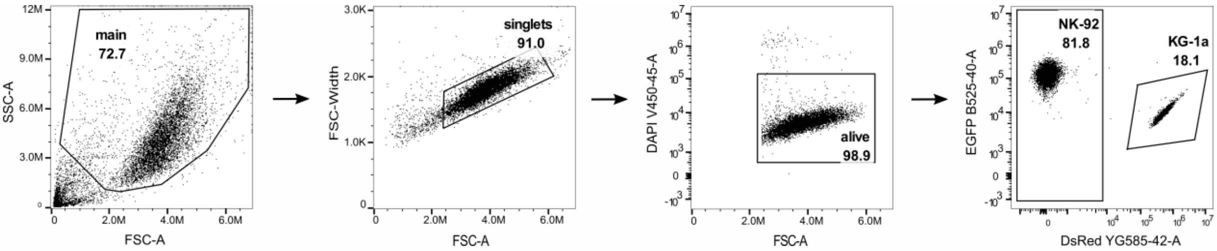

Figure S1. Representative dot plots showing a typical co-culture experiment. Cell populations were gated for singlets and vital cells were gated based upon the DAPI (4′,6-diamidino-2-phenylindole) negative sub-population according to cells that were killed by heating (90 °C, 5 minutes). NK-92 cells and KG-1a cells were separated based on EGFP and DsRed expression, respectively.
